# The association of COVID-19 nexus on China’s economy: A financial crisis or a health crisis?

**DOI:** 10.1371/journal.pone.0272024

**Published:** 2022-09-07

**Authors:** Cheng Hu, Wei Pan, Wulin Pan, Wan-qiang Dai, Ge Huang

**Affiliations:** 1 School of Economic and Management, Wuhan University, Wuhan, China; 2 School of Applied Economics, Renmin University of China, Beijing, China; Szechenyi Istvan University: Szechenyi Istvan Egyetem, HUNGARY

## Abstract

This paper analyses the interaction between the novel coronavirus pandemic (COVID-19), unemployment rate, stock market, consumer confidence index (CCI), and economic policy uncertainty (EPU) index in China within a time-frequency framework. We compare the changes in economic indicators during the global financial crisis (GFC) and study the different impacts of the two events on China’s economy. An unprecedented impact of COVID-19 shocks on the unemployment rate, CCI, EPU index, and stock market volatility over the low frequency bands is uncovered by applying the coherence wavelet method to China monthly data. The COVID-19 effect on the stock market volatility and the EPU index is substantially higher than on the unemployment rate and the CCI. On the contrary, the GFC’s impact on the unemployment rate is much greater than that on the EPU index and CCI. Additionally, the impact of the GFC on the economy is more cyclical in the long-term, while the COVID-19 pandemic is a short-term shock with a relatively short oscillation cycle. This study concludes that the economic impact of COVID-19 will not spread into a financial crisis for China and believe that the COVID-19 pandemic is more of a health event than an economic crisis for Chinese economy.

## Introduction

Since the 20th century, China has faced two serious shocks: the spread of the novel COVID-19 pandemic since December 2019 and the global financial crisis (GFC) in 2008. The two emergencies have initiated a sharp downturn for China’s economy. The former reflected a direct blow to China’s real economy. At the same time, the latter meant that the deep-rooted contradictions in China’s economic development were further magnified by the financial crisis [1]. During the GFC, the Chinese economy entered a period of adjustment under the influence of the twin economic transition and cyclical adjustment factors. This meant that the direct impact of GFC on China’s macro economy was minimal, while the indirect was relatively large [2]. As a result, China’s GDP growth rate fell from 10.6% in the first quarter to 9% in the third quarter of 2008 [3]. Additionally, the GFC indirectly affected China’s stock and housing markets, causing a drop in price and putting enormous pressure on the appreciation of the renminbi (RMB). Also, the GFC brought about a series of social impacts. For example, health inequalities increased in the wake of the financial crisis, especially those associated with employment status, age, and family type [4].

Coronaviruses are a diverse group of viruses infecting many different animals, they can cause mild to severe respiratory infections in humans [5]. The World Health Organization (WHO) officially declared COVID-19 a pandemic in March of 2020 [6]. Therefore, to cope with the pandemic, stay-at-home policies were implemented to slow the spread of COVID-19 [7]. The emergence of the epidemic and attempts to limit its spread resulted in a contraction of the global economy [8]. The Chinese economic policy uncertainty (EPU) index, as observed by Baker, S. R. and Bloom, N [9], jumped from 195 to 501 between February to May 2020. Jianlei Yang and Chunpeng Yangrevealed a significant additional increase in stock market volatility with a higher sensitivity to EPU index after the lockdown in China [10]. The epidemic also had a huge impact on China’s private enterprises. According to the analysis of a questionnaire survey of 1,000 enterprises conducted by China Entrepreneur magazine on "How much does the epidemic affect your business", COVID-19 generally affected various industries. About 21.70% of the enterprises surveyed believed that the manufacturing industry has suffered huge losses during the epidemic, accounting for the highest percentage. Furthermore, 15.80% of physical retail enterprises expected their losses to be significant, coming in second [11]. In comparison, the primary sector was relatively unaffected by the epidemic, compensating to a certain extent for the economic losses incurred by other sectors [12]. The impact on the tertiary sector varies, with accommodation and catering cultural, sports and entertainment businesses being the most affected and less adversely on internet-related services [13].

The COVID-19 pandemic is a source of systematic risk. Therefore, there is a need for further research on the economic effects of the coronavirus spread. Compared with the GFC, COVID-19 has a more tremulous global impact. This study specifically focuses on Chinese markets for several reasons. First, China was the first country to experience the epidemic outbreak. Therefore, Chinese authorities already had more information and experience of the risk related to the epidemic spread. Second, China was the first country to control the spread of COVID-19 and could help other countries mitigate the risk. Third, the Chinese people’s lives have returned to normal since March 2020 while other countries still engage in severe epidemic prevention. Thus, by analyzing the impact of the COVID-19 shock on the Chinese market, we can provide useful insights for the contagion and spillover effect studies in other countries and regions.

## Literature review

From the global economic perspective, it can be argued that global economic growth is facing the worst since the Great Depression of the 1930s. For the first time, developed and developing economies are in a recession. As the most effective means of controlling the spread of the epidemic was quarantine and containment, the government introduced a lockdown, causing unemployment to rise [14]. The government in general has also been under pressure due to the rapid increase in spending on epidemic prevention. The US labor unemployment rate reached at least 17%, surpassing the peak of 10.6% in 2008 and was the highest since the Great Depression in 1929. Global economic growth plummeted in the March quarter of 2020 due to lockdowns and social distancing policies [15]. Although economic resilience and other economic factors reduce market volatility [16], COVID-19 has caused significant volatility in global stock markets. Oil price has significantly affected the US stock market and the US dollar exchange rate [17]. While COVID-19 infections in Australia have a significant positive effect on the performance of the stock market: a one standard deviation increase in new confirmed cases of COVID-19 infections increases the daily growth rate of the ASX-200 by around half a percentage point [18]. Jan Jakub Szczygielski et al. use Google Trends search data as a proxy for capturing uncertainty associated with the COVID-19 pandemic and investigate its impact on global industry returns. The results show that all industries are negatively impacted by COVID-19 related uncertainty [19]. Global supply and industrial chains have been disrupted, with trade and manufacturing growth falling further [20]. Jan Jakub Szczygielski et al. argue that countries’ energy sectors further west from the virus outbreak are impacted to a greater extent by COVID-19 related uncertainty [21].

Since the epidemic outbreak, several research studies have investigated whether COVID-19’s disaster is more perilous than the GFC. Scholars prefer to use qualitative analysis to research the impact of COVID-19 and GFC on the economy and then draw relevant conclusions through comparative study. Most studies believe that the COVID-19 pandemic is more serious than the GFC and could trigger an economic crisis. Syed Kumail Abbas Rizvi and Rania Itani compare the dynamics of the oil market during COVID-19 crisis with that of during Global financial crisis of 2008 and the SARS outbreak of 2002–2004. The results confirm high negative skewness and high positive kurtosis during COVID-19, roughly 43 times worse than the SARS crisis and 23 times worse than the GFC [22]. Additionally, a high-fat tail risk implies that COVID-19 is a low probability but high severity event. Khurram Shehzad and Liu Xiaoxing et al. hold that COVID-19 has influenced the variance of the US, Germany, and Italy’s stock markets more than the GFC. However, the GFC showed a more significant impact on the financial volatility of the Nikkei 225 index and SSEC than COVID-19. The analysis authenticated that the health crisis due to COVID-19 instigated the global financial crisis [23]. Shehzad and Khurram show that the GFC positively affected oil returns, but COVID-19 had a negative effect. They confirmed that COVID-19 is a bigger crisis than the GFC and has critically disturbed the global financial and commodity markets [24]. There are also researches focusing on industrial reactions to the two events [25] or contrasts the financial system’s resilience during the GFC and COVID-19 [26]. However, some experts believe that the COVID-19 pandemic will have a limited impact and will not cause a large-scale economic crisis. Ippei Shibata uses the U.S. Current Population Survey data to compare the distributional impacts of the COVID-19 Pandemic Crisis and those of GFC in terms of worker employment. They claim that workers with low-income earnings have suffered more than top-income earners in both recessions, suggesting a significant distributional impact of the two recessions [27]. More importantly, many unemployed people were on temporary layoff during the COVID-19 recession, unlike the GFC.

This study focuses on the Chinese market and uses wavelet analysis method to study the correlation between these two events on economic indicators. First, we examine how China’s unemployment rate, EPU index and CCI changed during the global financial crisis as the financial market fluctuated. Similarly, we show that during the COVID-19 outbreak, the domestic unemployment rate, EPU index, stock price index (SPI), and the CCI has changed with the epidemic situation. Then, we use wavelet analysis to study the correlation between the indicators separately in the two events. Finally, McKee, M. and Stuckler, D. hold that the COVID-19 pandemic is a health crisis and rapidly becoming an economic one too [28]. Based on previous results, we compare the different impacts of the two events on China’s economic indicators and discuss whether the COVID-19 outbreak can be regarded as a huge economic crisis. The remainder of this paper is organized as follows. The next section explains the data and methodology employed in this study. Therefore, we present the empirical results. Finally, the last section concludes the study.

## Data and methodology

### Data and data resources

This study used data on monthly observations of COVID-19 counts (measured as the number of the confirmed infected cases in China), China-SPI as measured by the monthly closing price of Shanghai Composite Index, the unemployment rate in China, China-EPU (news-based index) [29] and CCI. We study these variables for three reasons: (1) the unemployment rate, CCI, SPI and EPU index can represent China’s macroeconomic performance; (2) the COVID outbreak first significantly impacted the market supply, affecting the unemployment rate and CCI, the stock market was plunged into wild swings; and (3) the objective of this study is to compare the impact of COVID-19 and the GFC on the macroeconomy. Both events have an impact on the above indicators for Chinese macroeconomy.

The COVID-19 data is collected from the National Health Commission of the People’s Republic of China website. Moreover, the CCI and the Chinese stock market data are collected from China Economic Information Network (CEI net) and EPS Data. The information about the EPU index is obtained from the website of EPU website. Data and data resources are presented in Table 1. This study identifies the start of the GFC as February 13rd, 2007, when New Century Financial Group issued its profit warning [30]. Considering that the end of the GFC is generally marked by the return of the Dow Jones Industrial Average to its pre-crisis level, the end date of the GFCs was set at December 31st, 2011. Additionally, to better analyze the medium to long-term impact of the COVID-19 on our economy, this study sets the time region of the COVID-19 event as January 2020 to October 2021. Thus, the time duration of collected monthly data is from February 2007 to December 2011 and January 2020 to October 2021 respectively, yielding 346 observations. The raw data are in S1 File. All the series are converted into natural logs during the empirical analysis.

**Table 1:** Data and data sources.

Event		Variable	Sources
COVID-19 2019.12–2021.7	Y	COVID-19 counts	http://www.nhc.gov.cn/
	X_1_	the unemployment rate	https://zh.tradingeconomics.com
	X_2_	China-EPU	http://www.policyuncertainty.com/
	X_3_	SPI	EPS Data
	X_4_	CCI	CEI net
GFC 2007.2–2011.12	Y	SPI	EPS Data
	X_1_	the unemployment rate	https://zh.tradingeconomics.com
	X_2_	China-EPU	http://www.policyuncertainty.com/
	X_3_	CCI	CE Inet

### Wavelet analysis and function definition

In time-series research, the time and frequency domains are two basic forms commonly used. Time-domain analysis has the function of time localization, but cannot generate more information about the time series change. Although frequency domain analysis (e.g., Fourier transform) has an accurate frequency location function, it is only suitable for stationary time series analysis. However, the change of many variables over time is often influenced by a variety of factors, and most of them are non-stationary series. They have tendency, periodicity, randomness, mutability, and "multi-time scale" structure characteristics, with multi-level evolution law. For the study of such non-stationary time series, time information corresponding to a certain frequency band or frequency domain information of a certain period is usually needed. On the other hand, wavelet analysis can reflect the trend and periodicity characteristics of interval changes.

Wavelet analysis has been gradually used to analyze economic and financial time series and their correlation in recent years. It can be used for filtering, denoising, multi-resolution time-frequency analysis, especially for non-stationary signal analysis. Currently, wavelet analysis is widely applied to the economy, such as renewable energy consumption and economic growth [31, 32], CO_2_ emissions and economic growth [33], and oil prices and economic fluctuations [34], etc. Ousama Ben-Salha et al. use the wavelet analysis and demonstrate that the relationship between the U.S. output and the aggregate energy used by source varies across frequencies and evolves over time [35]. Raza, S. A. et al. used wavelet coherence to analyze the relationship between oil prices and economic activity. The results indicate that oil prices have a positive impact on economic activity. Since the outbreak of COVID-19, scholars have also used wavelet analysis to study economic fluctuations [34]. Iqbal, N. et al. used the WTC methodology to analyze the nexus between weather, the COVID-19 outbreak in Wuhan and the Chinese economy. The results suggest that the RMB exchange rate and COVID-19 showed an out-phase coherence at specific time-frequency spots suggesting a negative but limited impact of the COVID-19 outbreak in Wuhan on the Chinese export economy [36]. Sunyong Choi applies wavelet coherence analysis to EPU data and monthly sector volatility of the S&P 500 index from January 2008 to May 2020. The results reveal that EPU regarding COVID-19 has influenced the sector volatility more than the GFC for all sectors [37].

All the integral is equal to the zero function can be called wavelet function, written as *ψ*(*t*). Its essence is to generate and decay in a finite period, and has the following two conditions:

1. *ψ*(*t*) is a function with finite support or zero velocity drop;
2.
$\int_{-\infty }^{+\infty }\psi \left(t\right)dt=0$

In practice, we usually study the signal *f*(*t*) with finite energy assuming the function *f*(*t*) ∈ *L*^2^(*R*), *L*^2^(*R*) represents the square integrable real number space, and ψ(*t*) ∈ *L*^2^(*R*) is also included. Therefore, to enable the wavelet transform to reconstruct the original signal, the wavelet function needs to meet the admissibility conditions:

$$C_{\psi }=\int_{0}^{+\infty }\frac{{\left|\hat{\psi }(w)\right|}^{2}}{w}dw<+\infty$$ (1)

A wavelet satisfying the admissibility condition is called an admissible wavelet. Wavelet transform is a family of wavelet functions obtained by stretching and translating ψ(*t*), which is also called fundamental wavelet or parent wavelet:

$$\left\{\psi_{a,b}\left(t\right)=\frac{1}{\sqrt{a}}\psi \left(\frac{t-b}{a}\right)|a>0,b\in R\right\}$$ (2)

Where, *a* is the expansion or scale factor, and *b* is the shift factor. When I changes, ψ_*a*,*b*_(*t*) can cover the different frequency ranges, and a smaller |*a*| corresponds to a higher frequency. When *b* changes, the centre of the time window moves with the time domain, thus achieving local localization in the time-frequency domain.

### Continuous wavelet transform

Continuous wavelet transform (CWT) expands the time series into time-frequency space. Assume the function *f*(*t*) ∈ *L*^2^(*R*), *ψ*(*t*) is an admissible wavelet, we call

$$\left(W_{f}\right)\left(a,b\right)=\int_{-\infty }^{+\infty }f\left(t\right)\frac{1}{\sqrt{a}}\overset{-}{\psi }\left(\frac{t-b}{a}\right)dt,a>0$$ (3)

as the continuous wavelet transform of the function *f*(*t*). Where *a* is the scale factor and *b* is the shift factor. $\overset{-}{\psi }(t)$ is the complex conjugate function of *ψ*(*t*).

### Wavelet coherence

Wavelet coherence (WTC) finds regions in time-frequency space where the two time-series co-vary (but do not necessarily have high power), like the Pearson product-moment correlation coefficient. According to the Fourier method, wavelet coherence is defined as the ratio of the cross spectrum to the product of the spectrum of each sequence. Therefore, wavelet coherence can be regarded as the local correlation of two time series in the time-frequency domain [38]. If the wavelet coherence is close to 1, there is a high correlation between time series, while if it is close to 0, there is no correlation.

Referring to Aguiar-Conraria and Soares [39], this study focuses on wavelet coherence because of the advantage of the wavelet phase in standard the power spectrum of two time series. Additionally, the wavelet cross spectrum still shows peak value even in the case of a pseudo-significance test of the independent process.

## Empirical results and discussion

### Trend analysis

**Fig 1:**
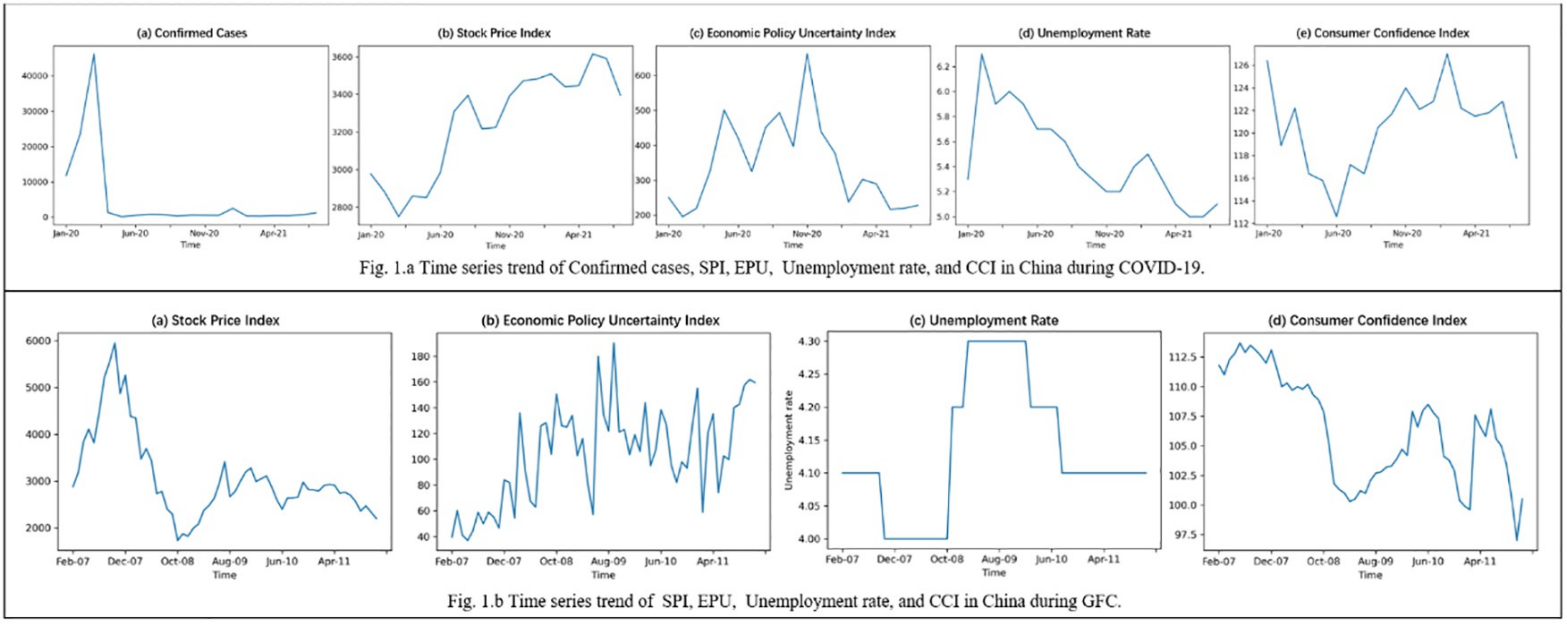
Time series trend of economic indicators in China during GFC and COVID-19.

Undoubtedly, news about the COVID-19 outbreak has been an important driver of market volatility in China. Fig 1 reports the economic indicators during COVID-19 (Fig 1a) and the GFC (Fig 1b), including financial markets measured by the SPI, EPU, CCI and unemployment rate. During the COVID-19 epidemic, the exponential rise in infections in the first quarter accompanied a precipitous drop in the SPI. However, as domestic infections cleared in April 2020, the SPI rapidly rose between April and August and increased at a moderate pace after reaching 3,400. By comparison, during the GFC, the domestic stock market plummeted from 2007 to 2008, dropping below 2,000 in October 2008. It then recovered to around 3,000 points for the following months. The State Council executive meeting set out ten measures to expand domestic demand and a $4 trillion investment plan in November 2008. The variance of SPI was 915.04 in the four years of GFC, approximately three times larger than that in the COVID-19 period with a range of 9 months. It shows a more violent fluctuation in the stock market during the GFC.

During the COVID-19 period, the EPU continued to increase after January 2020. However, even after the epidemic was controlled effectively, it reached 600 points in November 2020, probably related to the recurrent outbreaks of the epidemic in winter. After that, it began to decline gradually in 2021. Comparatively, the EPU fluctuated up and down between 40 and 180 during the GFC. The variance of the EPU through the 4-year period was 37.96, compared with 117.04 during the COVID-19 period of one and a half years, demonstrating greater economic policy volatility during COVID-19. As the unemployment rate shows, the lockdown policy at the beginning of January 2020 significantly lifted the rate. However, after March 2020, with the recovery from the epidemic, unemployment problems began to gradually alleviate, with the rate dropping to around 5% on June 21st and then stabilizing. During the GFC, the unemployment rate ranged from 4% to 4.3%.

Affected by the lockdown policy and people’s panic, CCI showed an obvious inverted ‘V’ shaped trend with a free fall until the end of June 2020, though infections in mainland China cleared in April. Thereafter, the index turned to a steady rise in the following six months. Comparatively, the CCI remained under 112 points during the GFC. The index began to decline at the end of 2007, dropping to 100 points around 2009, and showed signs of recovery after that, which may be a result of the nationwide value-added tax reform and consumption stimulation. However, the figure still experienced two significant falls around 2011, reaching a low of 97.5. The variance of CCI in COVID-19 and GFC were 3.88 and 4.50, meaning greater volatility of CCI in the GFC.

It shows that both the COVID-19 and the GFC posed a threat to the global economy. Unemployment rate volatility has been slightly more volatile during the GFC than during pandemics. The SPI and CCI’s volatility during the COVID-19 pandemic is much lower than during the GFC, while the EPU’s volatility is much higher. Since the COVID-19 seems to be in unstable condition, there is still considerable uncertainty about the future. These concerns have motivated our further study.

### The continuous wavelet transforms

**Fig 2:**
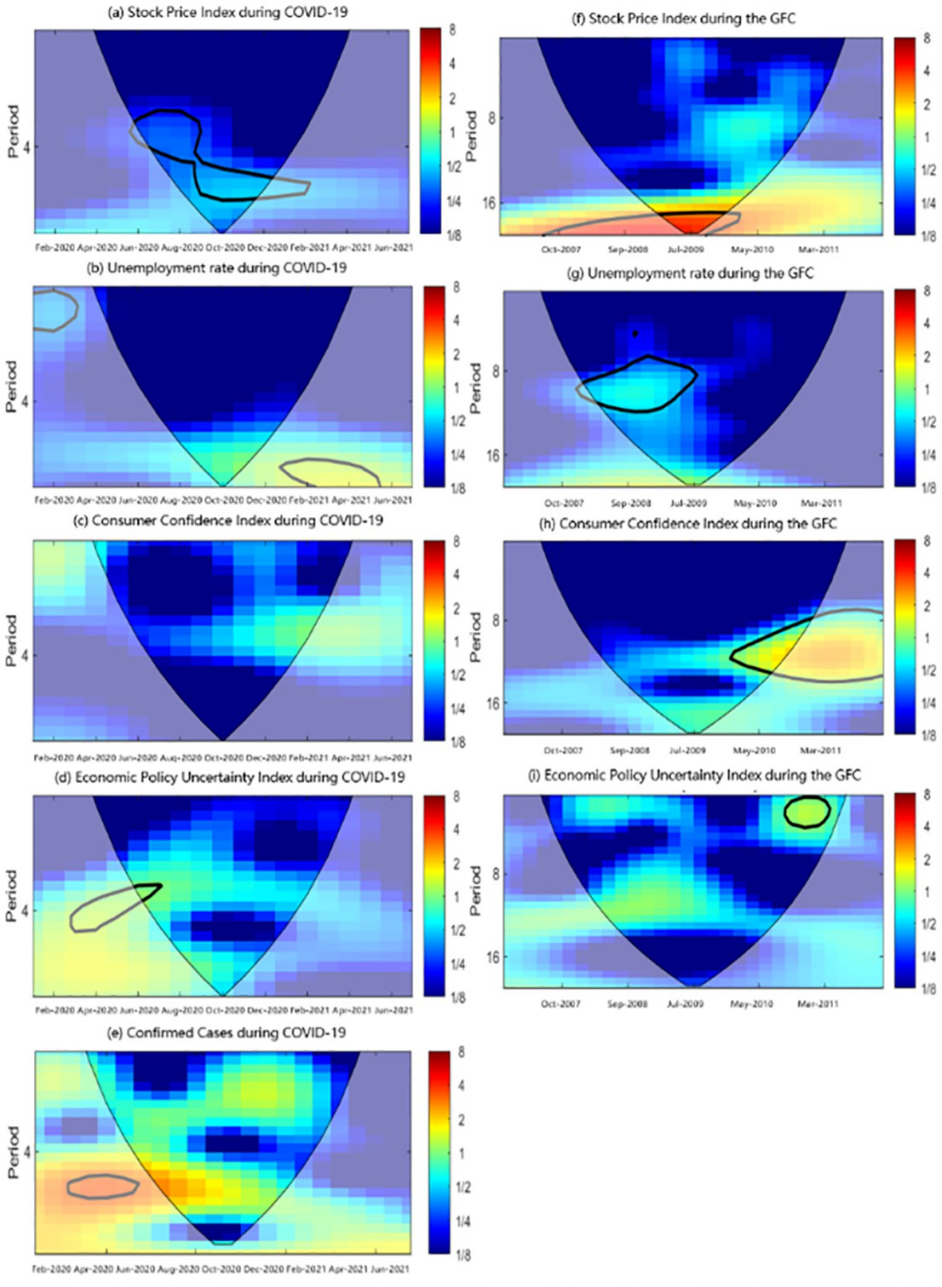
CWT plots for the SPI, EPU, CCI and unemployment rate during COVID-19 and GFC in China.

This study uses the Matlab2021 software for wavelet analysis. The CWT plots for each variable are conveyed in Fig 2. The CWT describes the movements of each variable in the time-scales and frequency bands. Where X-axis is time and Y-axis is cyclicality. The vertical color bar on the right side refers to the intensity of the period change, where red and blue correspond to high energy (high fluctuation) and low energy (low fluctuation) respectively. The colored region in the symmetrical curve is an effective periodic region of time-series variation. The redder the color in the power spectrum, the more severe fluctuation under time-frequency. The bluer color indicates that the time series has a small fluctuation range and is relatively stable. The bold black line in the figure represents an estimate of the 5% significance level from 1000 simulations using the Monte Carlo method. The area below the black line is the influence cone, representing the presence of edge effects. Wavelet energy spectrum is used as the index of the local sequence fluctuation. In the figure, period represents frequency and year represents time.

All economic indicators showed significant mid-to-high frequency fluctuations during COVID-19 (Fig 1a–1e). The number of confirmed cases showed significant medium-term to long-term high-frequency fluctuations from February to May 2020 (Fig 2e) with a cycle of 5–6 months. In February 2020, China’s National Health Commission implemented a series of measures to encourage residents to stay at home, including shutting down cities and roads. Local party members and cadres, and grassroots medical workers conducted household surveys in communities, quickly revealing the development of the epidemic in China. In February 2020, the daily number of new confirmed cases jumped to its zenith. In April 2020, the epidemic was basically under control in China and social activities gradually returned to normal. Until June 2020, when the epidemic re-emerged in a few regions, all regions could respond quickly to control the situation based on previous experience. Therefore, the daily number of newly confirmed cases remained low. The development of the epidemic directly led to the medium to high-frequency fluctuation of the EPU from February to July 2020 (Fig 2d), which is in line with the time trend shown in Fig 1. From May 2020 to March 2021, the stock market shows a medium-term with a low-frequency fluctuation state (Fig 2a). Due to the increasingly severe global epidemic situation and the impact of the oil price war, the global financial market experienced a shock in the first quarter. On March 9th, 12th, 16th, and 18th, 2020, the S&P 500 index fell by more than 7% in intraday trading, causing the stock market’s circuit breaker mechanism for four times. However, under China’s state control, the Central bank adopted a relatively loose monetary policy, alleviating the liquidity crisis and the epidemic’s impact o, such that the SPI did not fluctuate significantly. The unemployment rate showed short-term and low-frequency fluctuations during February and March 2020, and transferred to medium-term with high-frequency fluctuations from February to May 2021 (Fig 2b). During the epidemic, most companies suffered a financial crisis, a capital chain of enterprises was broken, and some companies even went bankrupt and were liquidated. The domestic unemployment rate fluctuated and briefly rose. At the beginning of 2021, as the epidemic situation in China further stabilized, the economic situation improved, and the unemployment rate showed a downward trend. However, there was no significant fluctuation in CCI (Fig 2c). During the study period, CCI showed an obvious inverted ‘V’ shaped trend. From January 2020 to July 2020, CCI continued to decline and then rose steadily, but the wavelet transform results do not show obvious cyclical fluctuations.

During the GFC, the SPI had sustained long-term and high-frequency fluctuations from August 2007 to April 2010 (Fig 2f), during which the stock market declined. From November 2007 to July 2009, the unemployment rate showed significant medium to high-frequency fluctuation (Fig 2g), and the unemployment rate gradually increased in the fluctuation. From March 2010 to the end of 2011, the CCI showed long-term significant high-frequency fluctuations (Fig 2h), and experienced two rising and falling processes. The EPU showed significant short-term and low-frequency fluctuation from June 2010 to May 2011 (Fig 2i).

Table 2 compares the fluctuations of the four economic indicators during the COVID-19 pandemic and the GFC. The comparison shows that economic indicators have more obvious periodic characteristics with longer cyclicity during the GFC. At the same time, they have more volatility with shorter cyclicity during the COVID-19 pandemic. This study holds that the two aspects contribute to the phenomenon: The first is the difference in the research time limitation of the two events. The study period of COVID-19 was 22 months, while the GFC lasted 59 months. Therefore, the GFC has an advantage in cyclical characteristics. The second is the difference in the mechanism of events. The GFC in 2008 was triggered by the expansion of the US real estate mortgage market, which led to the excessive issuance of US currency and excess liquidity of US trading partners. It was also the crisis of confidence in US dollar caused by liquidity depletion of the global financial market. Finally, the global spread of the epidemic triggered the COVID-19 crisis in 2020. The tipping point was the collapse of global capital markets, a wave of business failures, and job losses.

**Table 2:** CWT plots comparison of the COVID-19 and the GFC.

	Cyclicity		Frequency	
	COVID-19	The GFC	COVID-19	The GFC
SPI	Mid	Long	Low	High
Unemployment Rate	Short to Medium	Long	Low to Medium	Low
CCI	-	Long	-	High
EPU	Mid	Short	High	Low

Note: This study takes the cycle characteristics of less than 4 months as short-term, 8 months as medium-term cycle, and more than 16 months as long-term cycle by considering the difference in the time span of the two events studies and the data analysis results, referring to relevant literature.

Our comparison of the causes of the two crises established that the occurrence of the GFC was due to the inherent problems in the American financial system. The financial liquidity crisis caused the failure of financial institutions, thus causing the long-term fluctuations of various economic indicators. On the other hand, the COVID-19 crisis was a crisis in the real economy caused by the lockdown. Although the stock market fluctuated during the epidemic, domestic social activities quickly recovered after the epidemic was brought under control. Therefore, various economic indicators quickly stabilized after the short-term fluctuations. However, the epidemic crisis is likely to evolve into a financial crisis and further trigger an economic crisis if the liquidity crisis and the decline in investor confidence caused by the epidemic cannot be quickly recovered under policy adjustment, and the epidemic changes seriously hinder the resumption of work and production, the deterioration of corporate bonds leads to the bursting of the fragile capital market bubble. Therefore, this study argues that the economic impact of COVID-19 will not spread into a financial crisis for China.

### The wavelet coherence

**Fig 3:**
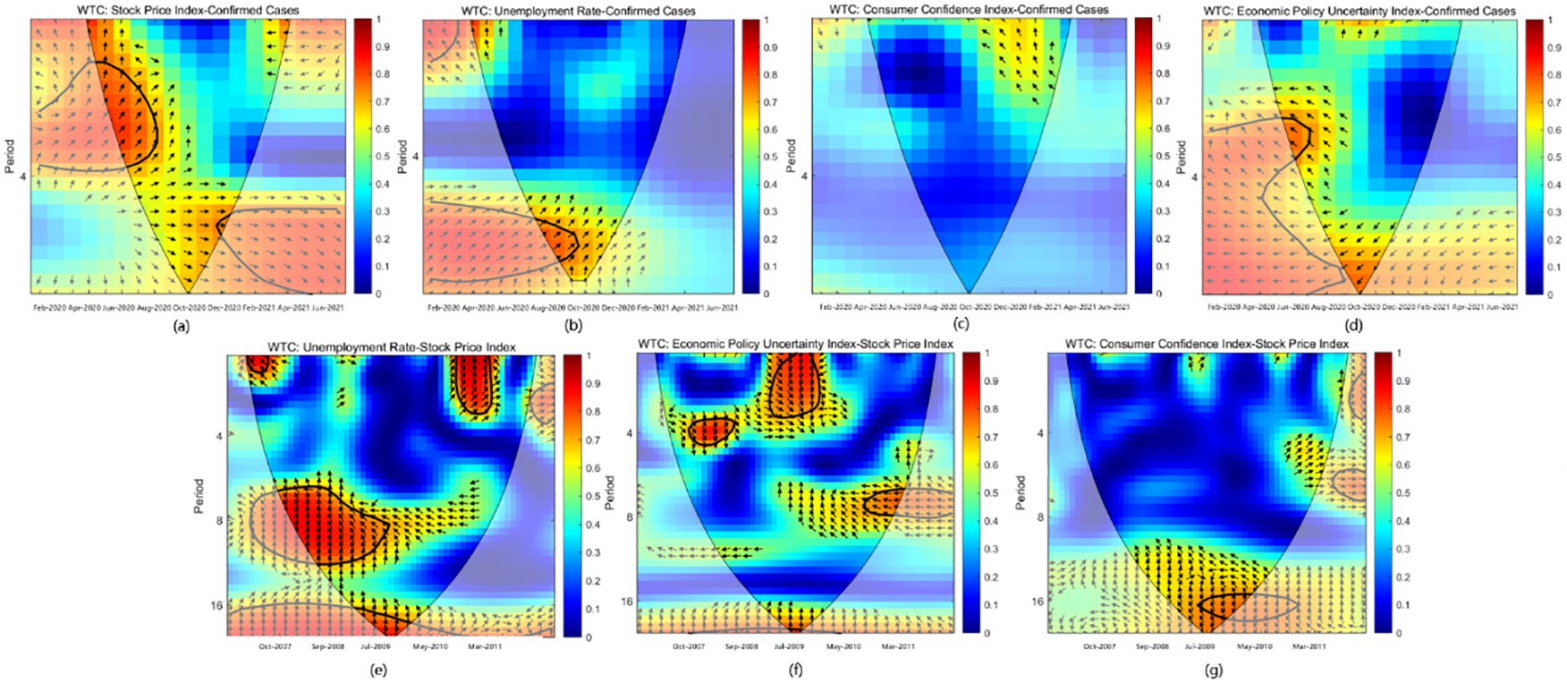
Wavelet coherence plots, pairwise estimates. Notes: The X-axis represents the time whereas, the Y-axis shows the period (in months).

For the study of causality between bivariate economic time series, the existing theoretical framework includes an econometric model, spectral analysis, wavelet analysis, etc. However, only the relationship between short-term and long-term variables can be analyzed when an econometric model is used solely to analyze the causal relationship between bivariate economic time series. The change in the relationship between variables cannot be further analyzed from the more detailed fluctuation period. Spectrum analysis can analyze the periodicity of variable fluctuation from the frequency domain perspective, but it lacks the specific location in time. In recent years, the theoretical framework of wavelet analysis has been applied to economic and financial time series analysis, which can be used to analyze the relational characteristics of bivariate economic time series under different fluctuation periods. Therefore, this study further analyzed the periodicity and correlation characteristics between indices and obtained the wavelet coherence graph between indices (Fig 3), The indices analyzed the relationship between pairs of variables, but also pointed out the direction of coherence on the time-frequency scale.

The arrow in the figure indicates the phase relationship of the two indexes. The arrow pointing to the right indicates the positive phase; the arrow pointing vertically upward indicates that the wavelet changes of variable 2 advances variable 1 by about 1/4 cycle; the arrow pointing to the upper left 45 degrees represents that variable 2 advances variable 1 by about 1/8 cycle, indicating that the change of variable 2 has a certain promoting effect on the occurrence of variable 1. The arrow pointing 45 degrees to the upper right means that variable 2 is 1/2 cycle ahead of variable 1, and vice versa.

Fig 3a–3d show the relationship between confirmed cases and economic indicators during the COVID-19. We identify a quite similar configuration in islands of strong coherencies in Fig 3a, showing the wavelet coherence between the SPI and COVID-19 Outbreak. It indicates a strong dependency over the 1 to 4-months frequency bands from February 2020 to August 2020. The number of confirmed cases is about 1/2 cycle ahead of the SPI, indicating that the increase in the number of confirmed cases has a short-term promotion effect on the SPI volatility. Unsurprisingly, the arrows are predominantly turned up and to the right, indicating that Confirmed Cases are leading the Chinese stock market. From December 2020 to July 2021, the number of newly confirmed cases lagged the SPI cycle by about 1/8 with a cycle of 4–7 months. Nevertheless, the two indicators still positively affect each other, the volatility sensitivity of SPI decreases, and the cycle increases.

The unemployment rate and CCI are lagging indicators relative to the number of newly confirmed cases. Fig 3b shows the wavelet plot between China’s unemployment rate and COVID-19 counts. From February to March 2020, the number of new confirmed cases had a short-term negative effect on the unemployment rate. The number of new confirmed cases was about 1/8 cycles ahead of the unemployment rate. We identify an island of high dependence over the 5–8 months frequency band from February 2020 to October 2020 and the arrows are mostly pointed up to the right. Other coherencies over 1–2 months’ frequency bands, with arrows turned up and to the right, suggest a cyclic relationship between COVID-19 and the unemployment rate. These results show that the COVID-19 pandemic seems to severely impact unemployment rate volatility on the production side because of travel restrictions and low expected output growth in China. The number of newly confirmed cases rose sharply in the first two months and then gradually decreased. As a lagging indicator, the unemployment rate gradually decreased in the post-epidemic period.

As for the connectedness between the COVID-19 Outbreak and CCI (Fig 3c), the WC plot reveals the light existence of coherency islands over the research period. The arrows are mostly turned up and to the left, implying that the COVID-19 Counts are leading the CCI and the sharp decrease in the counts has strikingly raised the CCI in China. The EPU-Confirmed Cases connectedness over time-scales and frequencies is shown in Fig 3d. The EPU is strongly affected by COVID-19 Outbreak. Indeed, a strong island of red color is identified from early Feb-2020 to Oct-2020, with a frequency band of 3 to 8 months, corresponding to the unexpected oil market and the increasingly serious epidemic situation abroad. Additionally, the arrows are mostly turned down to the left, implying an anti-cyclic relationship between the EPU and COVID-19 Counts, with the former lagging behind the taper count.

Fig 3e–3g show the relationship between SPI and economic indicators during the GFC. The wavelet plot between Unemployment Rate and SPI are presented in Fig 3e. The dependence structure is principally observed over the long-run (up to 16-months-time horizons), and the arrows are mainly turned up and the right, showing the SPI is the leading variable. The unprecedented volatility jumps in the SPI are raising the unemployment rate in China.

As for the dependence between the EPU and SPI (Fig 3f), the wavelet plot is extremely edifying. We identify areas of strong dependence from the inception to the end of the period. For the entire period, arrows are predominantly turned up, revealing the SPI as the leader and the EPU as the follower. In 2008, the influence of the two indicators was in the same direction in the short term from 1 to 4 months, but in 2009, it was in the opposite direction with medium-term cyclicity. We believe that the rising volatility of the stock market stimulates the rise of EPU. However, in 2011, the SPI increase caused an EPU decline in the late period of the GFC. This is the phenomenon of the stock market turning around after the economic situation improves and EPU is relatively stable. Nonetheless, people’s panic about the GFC is the main driver of the EPU and seems to harm the chances of international cooperation to overcome the GFC.

CCI is an obvious lagging indicator. In the long run, SPI leads CCI by about 1/4 cycles, and the two indicators promote each other in the short run. The wavelet coherencies between the CCI and SPI are conveyed in Fig 3g. This figure shows the existence of small hot red zones in almost 16 months frequency band (i.e. long-time horizons), and there is a strong dependence between the two variables. For these small zones, arrows are mostly turned right, indicating the same direction. In summary, the SPI-CCI relationship and causalities vary through time-scales and frequency banks.

By comparison, it can be found that the cycle characteristics of the GFC are obvious, and most of them are medium to long-term effects, with relatively weak impact. The impact of COVID-19 is more significant in the short to medium term. The COVID-19 pandemic has severely impacted the real economy, leading to a sharp rise in pessimistic expectations from various sectors. The 21th century has yet to see a pandemic like COVID-19, whose contagiousness and ubiquity have once terrified countries. In the short term, the normal production of society is blocked, and even the production development is suspended. Firstly, people’s normal activities are restricted due to home isolation. Secondly, the supply chain is blocked, causing temporary production shortages. Additionally, a series of capital flow fracture problems are caused by operating pressure and inventory retention. However, in the long run, countries quickly adopted epidemic prevention measures suited to their national conditions, mobilized massive human and material resources to overcome the epidemic, gradually controlled the epidemic, and people’s production and life quickly resumed. Therefore, in the long run, the economic impact of COVID-19 will be gradually diluted, and the national economy will quickly return to normal levels.

We hold that the economic impact of the COVID-19 pandemic is more short-term, although significant, but of short duration. On the contrary, since the GFC originated from the subprime crisis, it shocked the entire international financial market. Also, it had a longer lasting impact on China’s economy and even the whole world, but the impact was less pronounced. The root causes of the GFC were the excessive leverage ratio of the household sector, the continuous defaults of the subprime loan market, and the bankruptcy of financial institutions. Before the GFC, China’s dependence on foreign trade increased from 38.5% in 2001 to 64.8% in 2007. Affected by the GFC in 2008, the dependence on foreign trade decreased to about 60%. The GFC triggered a serious economic recession, putting China’s trade balance under severe test and greatly impacting investment, consumption, and employment. Due to the sluggish economic environment at home and abroad, sluggish stock market, declining economic performance and rising unemployment, people’s income expectations have been lowered, consumption growth has begun to slow down, and the economy’s vitality has been reduced. As a result, the GFC has a longer impact on the economy.

## Conclusions

This study uses the wavelet method to analyze the impact of COVID-19 and the GFC on various economic indicators in China, including the stock market, the domestic unemployment rate, the EPU index and the CCI. Additionally, we discuss whether COVID-19 can induce a financial crisis. So far, only short period data on the pandemic can be collected. However, estimating the current and the future time-frequency causalities between COVID-19 cases, EPU, stock markets, and consumer reactions requires long period data to get high statistical inferences from the tests applied in this study. Therefore, most of the traditional econometric techniques are not appropriate for the current study. Furthermore, the wavelet transform overcomes the shortcomings of the Fourier transform where instantaneous changes in the time domain are not reflected in the frequency domain. As a result, it has a high-frequency resolution and low time resolution in the low-frequency part and a high time resolution and low-frequency resolution in the high-frequency part [40]. Therefore, this study uses the wavelet method since it is not affected by the sample size.

Through time trend analysis, wavelet transform analysis and wavelet coherence analysis, we concluded that SPI and CCI fluctuated greatly during the financial crisis. In contrast, the EPU fluctuated more sharply during the COVID-19 pandemic. Unemployment fluctuates in a very similar way. In terms of the wavelet transform, the comparison shows that economic indicators showed more obvious periodic characteristics with longer cyclicity during the GFC. In comparison, the COVID-19 pandemic showed more volatility with shorter cyclicity. Moreover, the cycle characteristics of the GFC are obvious, and most have medium to long-term effects with relatively weak impact. The impact of COVID-19 is more significant in the short to medium term. Therefore, this study argues that the economic impact of COVID-19 will not spread into a financial crisis for China. We believe the COVID-19 pandemic is more of a health event than an economic crisis for Chinese economy.

The number of confirmed COVID-19 cases continues to rise, and the virus continues to mutate. The global epidemic is far from over, and the risk of a new epidemic outbreak always exists. COVID-19 can exacerbate pre-existing health problems, with significant knock-on effects on socio-economic pressures and other aspects of healthcare systems. Therefore, it is more necessary to study and monitor the impact of COVID-19 on key socio-economic indicators. Based on existing economic indicators, this study concludes that COVID-19 has a greater impact on China’s economy with short-term cyclicity, while the impact cycle of the GFC is longer. Therefore, we believe that COVID-19 is a health crisis that has not induced an economic crisis. However, this study has limitations: Firstly, the representativeness of indicators is insufficient. The economic impact involves investment, consumption, import and export, and other aspects, and the economic indicators in this study are not enough to cover all economic activities. A more complete economic indicator system or even economic model can be constructed in future studies to summarize the influence situation in detail. Secondly, the study’s conclusion classifies COVID-19 as a health event, but this study has not compared it with other health events. Strictly speaking, the study could only confirm that COVID-19 does not fit into an economic crisis. Finally, different emergencies have diverse risk characteristics. We can further classify emergencies reasonably, and then study the various impact mechanisms of disparate kinds of emergencies on China’s economy.

## Supporting information

S1 File (DOCX)
