# Supplementary material for: The association of COVID-19 nexus on China’s economy: A financial crisis or a health crisis?
Source: PLoS One. 2022 Sep 7;17(9):e0272024. doi: 10.1371/journal.pone.0272024 (PMC9451089; doi:10.1371/journal.pone.0272024)
Supplement: S1 File — (DOCX) [file pone.0272024.s001.docx]

**Supporting Information**

**Supporting Table 1 Data of the COVID-19**

| **Time** | **Confir** | **Unemp** | **EPU** | **stock** | **CCI** |
| --- | --- | --- | --- | --- | --- |
| Dec-19 | 0 | 5.2 | 337.92147 | 2584.57 | 126.6 |
| Jan-20 | 11791 | 5.3 | 250.3219 | 2976.53 | 126.4 |
| Feb-20 | 23538 | 6.3 | 195.70617 | 2880.3 | 118.9 |
| Mar-20 | 46225 | 5.9 | 219.40251 | 2750.3 | 122.2 |
| Apr-20 | 1320 | 6 | 328.8054 | 2860.08 | 116.4 |
| May-20 | 143 | 5.9 | 501.33444 | 2852.35 | 115.8 |
| Jun-20 | 517 | 5.7 | 421.64111 | 2984.67 | 112.6 |
| Jul-20 | 803 | 5.7 | 324.66257 | 3310.01 | 117.2 |
| Aug-20 | 721 | 5.6 | 451.63109 | 3395.68 | 116.4 |
| Sep-20 | 356 | 5.4 | 493.40204 | 3218.05 | 120.5 |
| Oct-20 | 583 | 5.3 | 396.79675 | 3224.53 | 121.7 |
| Nov-20 | 545 | 5.2 | 661.82805 | 3391.76 | 124 |
| Dec-20 | 529 | 5.2 | 439.12364 | 3473.07 | 122.1 |
| Jan-21 | 2493 | 5.4 | 378.35705 | 3483.07 | 122.8 |
| Feb-21 | 348 | 5.5 | 237.40861 | 3509.08 | 127 |
| Mar-21 | 305 | 5.3 | 302.30058 | 3441.91 | 122.2 |
| Apr-21 | 454 | 5.1 | 289.63003 | 3446.86 | 121.5 |
| May-21 | 451 | 5 | 216.86056 | 3615.48 | 121.8 |
| Jun-21 | 670 | 5 | 219.7628 | 3591.2 | 122.8 |
| Jul-21 | 1213 | 5.1 | 227.92451 | 3397.36 | 117.8 |
| Aug-21 | 1893 | 5.1 | 294.8278 | 3543.94 | 117.5 |
| Sep-21 | 1264 | 4.9 | 274.95002 | 3568.17 | 121.2 |
| Oct-21 | 1081 | 4.9 | 248.47364 | 3547.34 | 121.8 |

**Supporting Table 2 Data of the GFC**

| **Time** | **SPI** | **Unemp** | **EPU** | **CCI** |
| --- | --- | --- | --- | --- |
| Feb-07 | 2881.07 | 4.1 | 39.755437 | 111.8 |
| Mar-07 | 3183.98 | 4.1 | 60.24591 | 111 |
| Apr-07 | 3841.27 | 4.1 | 41.061601 | 112.3 |
| May-07 | 4109.65 | 4.1 | 37.013331 | 112.8 |
| Jun-07 | 3820.7 | 4.1 | 44.436444 | 113.7 |
| Jul-07 | 4471.03 | 4.1 | 58.835016 | 112.9 |
| Aug-07 | 5218.83 | 4.1 | 50.138049 | 113.5 |
| Sep-07 | 5552.3 | 4.1 | 59.033218 | 113.1 |
| Oct-07 | 5954.77 | 4 | 55.082712 | 112.6 |
| Nov-07 | 4871.78 | 1 | 46.671065 | 112 |
| Dec-07 | 5261.56 | 1 | 84.050545 | 113.1 |
| Jan-08 | 4383.39 | 4 | 82.042925 | 111.6 |
| Feb-08 | 4348.54 | 4 | 54.385758 | 110 |
| Mar-08 | 3472.71 | 4 | 135.94651 | 110.3 |
| Apr-08 | 3693.11 | 4 | 91.602679 | 109.7 |
| May-08 | 3433.35 | 4 | 67.599629 | 110 |
| Jun-08 | 2736.1 | 4 | 62.944362 | 109.8 |
| Jul-08 | 2775.72 | 4 | 126.04434 | 110.2 |
| Aug-08 | 2397.37 | 4 | 128.41674 | 109.3 |
| Sep-08 | 2293.78 | 4 | 103.93305 | 108.9 |
| Oct-08 | 1728.79 | 4 | 150.53025 | 107.9 |
| Nov-08 | 1871.16 | 4.2 | 126.0784 | 105.2 |
| Dec-08 | 1820.81 | 4.2 | 125.14151 | 101.8 |
| Jan-09 | 1990.66 | 4.2 | 133.94778 | 101.3 |
| Feb-09 | 2082.85 | 4.3 | 102.87419 | 101 |
| Mar-09 | 2373.21 | 4.3 | 116.05141 | 100.3 |
| Apr-09 | 2477.57 | 4.3 | 81.103881 | 100.5 |
| May-09 | 2632.93 | 4.3 | 57.258195 | 101.2 |
| Jun-09 | 2959.36 | 4.3 | 179.84662 | 101 |
| Jul-09 | 3412.06 | 4.3 | 134.78489 | 102.1 |
| Aug-09 | 2667.75 | 4.3 | 121.93505 | 102.7 |
| Sep-09 | 2779.43 | 4.3 | 190.3161 | 102.8 |
| Oct-09 | 2995.85 | 4.3 | 121.25445 | 103.2 |
| Nov-09 | 3195.3 | 4.3 | 123.05994 | 103.3 |
| Dec-09 | 3277.14 | 4.3 | 103.58065 | 103.9 |
| Jan-10 | 2989.29 | 4.3 | 119.16204 | 104.7 |
| Feb-10 | 3051.94 | 4.2 | 106.04946 | 104.2 |
| Mar-10 | 3109.11 | 4.2 | 143.92238 | 107.9 |
| Apr-10 | 2870.61 | 4.2 | 95.042161 | 106.6 |
| May-10 | 2592.15 | 4.2 | 106.92515 | 108 |
| Jun-10 | 2398.37 | 4.2 | 138.49051 | 108.5 |
| Jul-10 | 2637.5 | 4.2 | 127.50131 | 107.8 |
| Aug-10 | 2638.8 | 4.1 | 95.159783 | 107.3 |
| Sep-10 | 2655.66 | 4.1 | 82.114615 | 104.1 |
| Oct-10 | 2978.84 | 4.1 | 97.899683 | 103.8 |
| Nov-10 | 2820.18 | 4.1 | 93.287463 | 102.9 |
| Dec-10 | 2808.08 | 4.1 | 125.76594 | 100.4 |
| Jan-11 | 2790.69 | 4.1 | 155.04626 | 99.9 |
| Feb-11 | 2905.05 | 4.1 | 59.007437 | 99.6 |
| Mar-11 | 2928.11 | 4.1 | 120.77013 | 107.6 |
| Apr-11 | 2911.51 | 4.1 | 135.17174 | 106.6 |
| May-11 | 2743.47 | 4.1 | 74.122883 | 105.8 |
| Jun-11 | 2762.08 | 4.1 | 102.65471 | 108.1 |
| Jul-11 | 2701.73 | 4.1 | 99.841595 | 105.6 |
| Aug-11 | 2567.34 | 4.1 | 140.26476 | 105 |
| Sep-11 | 2359.22 | 4.1 | 142.51499 | 103.4 |
| Oct-11 | 2468.25 | 4.1 | 157.72154 | 100.5 |
| Nov-11 | 2333.41 | 4.1 | 161.85022 | 97 |
| Dec-11 | 2199.42 | 4.1 | 159.6144 | 100.5 |
